# Acute Care of Older Adults

**DOI:** 10.1093/geroni/igaf122.2000

**Published:** 2025-12-31

## Abstract

In this session the researchers will share their insights into the challenges of research in the acute care setting and their research outcomes.

